# Fatty acid activated PPARγ promotes tumorigenicity of prostate cancer cells by up regulating *VEGF* via PPAR responsive elements of the promoter

**DOI:** 10.18632/oncotarget.6975

**Published:** 2016-01-22

**Authors:** Farzad S. Forootan, Shiva S. Forootan, Xiaojun Gou, Jin Yang, Bichong Liu, Danqing Chen, Majed Saad Al Fayi, Waseem Al-Jameel, Philip S. Rudland, Syed A. Hussain, Youqiang Ke

**Affiliations:** ^1^ Molecular Pathology Laboratory, Department of Molecular and Clinical Cancer Medicine, Liverpool University, Liverpool L69 3GA, United Kingdom; ^2^ Antibiotics Research and Re-evaluation Key Laboratory, Sichuan Antibiotics Industrial Institute, Chengdu University, Chengdu 610081, China; ^3^ Urology Department of Affiliated Hospital, Chengdu University, Chengdu 610081, China; ^4^ Department of Pharmacology, School of Pharmacology and Bioengineering, Chengdu University, Chengdu 610081, China; ^5^ Department of Biochemistry, Liverpool University, Liverpool L69 3GA, United Kingdom

**Keywords:** prostate cancer, FABP5, PPARγ, PPRE, VEGF

## Abstract

In previous work, it is suggested that the excessive amount of fatty acids transported by FABP5 may facilitate the malignant progression of prostate cancer cells through a FABP5-PPARγ-VEGF signal transduction axis to increase angiogenesis. To further functionally characterise the FABP5-PPARγ-VEGF signal transduction pathway, we have, in this work, investigated the molecular mechanisms involved in its tumorigenicity promoting role in prostate cancer. Suppression of *PPARγ* in highly malignant prostate cancer cells produced a significant reduction (up to 53%) in their proliferation rate, invasiveness (up to 89%) and anchorage-independent growth (up to 94%) *in vitro*. Knockdown of *PPARγ* gene in PC3-M cells by siRNA significantly reduced the average size of tumours formed in nude mice by 99% and tumour incidence by 90%, and significantly prolonged the latent period by 3.5 fold. Results in this study combined with some previous results suggested that FABP5 promoted VEGF expression and angiogenesis through PPARγ which was activated by fatty acids transported by FABP5. Further investigations showed that PPARγ up-regulated VEGF expression through acting with the PPAR-responsive elements in the promoter region of *VEGF* gene in prostate cancer cells. Although androgen can modulate *VEGF* expression through Sp1/Sp3 binding site on VEGF promoter in androgen-dependent prostate cancer cells, this route, disappeared as the cells gradually lost their androgen dependency; was replaced by the FABP5-PPARγ-VEGF signalling pathway. These results suggested that the FABP5-PPARγ-VEGF signal transduction axis, rather than androgen modulated route, may be a more important novel therapeutic target for angiogenesis-suppression treatment of castration resistant prostate cancer.

## INTRODUCTION

Prostate cancer is a serious health threat to man, particularly in the developed countries. High dietary fat intake has been shown to have a significant correlation with a higher risk of prostate cancer [1] and high levels of trans-isomers of oleic and linoleic acids (long chain fatty acids) in blood are associated with an increased risk of prostate tumours [2]. Fatty acids are not only sources of energy in cells, but are also signalling molecules involved in metabolic regulation. Their regulatory effects on enzymatic and transcriptional networks can lead to regulations in gene expression, cell growth and survival pathways and inflammatory responses [3]. Fatty acid binding proteins (FABPs) are known as intracellular chaperons of lipids. They reversibly bind hydrophobic ligands such as saturated and unsaturated fatty acids with high affinity and transport them into the cells [4]. FABP5 is a 15 kDa cytosolic protein which belongs to the FABP family [5]. In addition to the skin, FABP5 is detected in endothelial cells of placenta, heart, skeletal muscle, small intestine, renal medulla and in Clara and goblet cells of lung [6]. Apart from prostate cancer, *FABP5* has also been implicated in malignancies of bladder, pancreas [7, 8], breast [9] and glioblastoma [10].

Previous studies demonstrated that FABP5 is overexpressed in malignant prostate and breast cell lines compared to their benign counterparts and the increased level of FABP5 can induce metastasis *in vivo* [11]. Further investigations revealed that metastasis-inducing activity of FABP5 was achieved by up-regulating *VEGF* [12]. Thus suppression of *FABP5* expression in a highly malignant prostate cancer cell line PC3-M significantly reduced their invasiveness *in vitro* [13] and inhibited their tumorigenicity *in vivo* by reducing the level of VEGF and microvessel densities. In contrast, increasing *FABP5* expression in the weakly malignant prostate cancer cell line LNCaP promoted their invasiveness and proliferation rate *in vitro* and increased their tumorigenicity *in vivo* [14]. Higher levels of both nuclear and cytoplasmic FABP5 in prostate carcinoma tissues are significantly associated with a reduced patient survival [15]. Recently, it was established that cancer promoting activity of FABP5 is closely related to its ability to bind and transport extracellular fatty acids to their nuclear receptors in prostate cancer cells [14].

Fatty acid receptors termed peroxisome proliferator-activated receptors (PPARs) belong to the nuclear hormone receptor superfamily of ligand-inducible transcription factors [16]. All three isotypes (PPARα, PPARβ/δ and PPARγ) have been shown to modulate lipid metabolism [17]. The important role of PPARs in carcinogenesis was highlighted by the ability of their ligands to affect cellular proliferation and differentiation or to interfere in apoptosis and angiogenesis. While different subtypes of PPARs may have effect on tumorigencity of different cancer types, high level of expression of PPARγ has been detected in prostate cancer and cancers of some other organs [18, 19]. Although it has been suggested that the increased FABP5 may interact with the increased level of PPARγ in a coordinated way to facilitate malignant progression of prostate cancer cells [20], the exact role of PPARγ in tumorigenicity of prostate cancer is not clear. Large amount of fatty acids transported by FABP5 can stimulate PPARγ [14], but how the activated PPARγ can increase the level of *VEGF* is not known. PPARs can regulate gene expression by binding to the PPAR responsive elements (PPRE) within the enhancer or promoter sites of the target genes. Although *VEGF* promoter region does contain several PPRE sequences, it was not known whether PPARγ can promote VEGF expression through binding to the PPREs in its promoter region to activate mRNA transcription.

In this work, experiments have been performed to study the molecular mechanisms of how FABP5 (or fatty acids transported by FABP5) transduces signals that eventually lead to an involvement in increased VEGF and facilitated malignant progression of prostate cancer cells in both androgen-dependent and particularly in androgen-independent subtypes.

## RESULTS

### Increased PPARγ expression produced by FABP5 and establishment of PPARγ-suppressed transfectants

**Figure 1:**
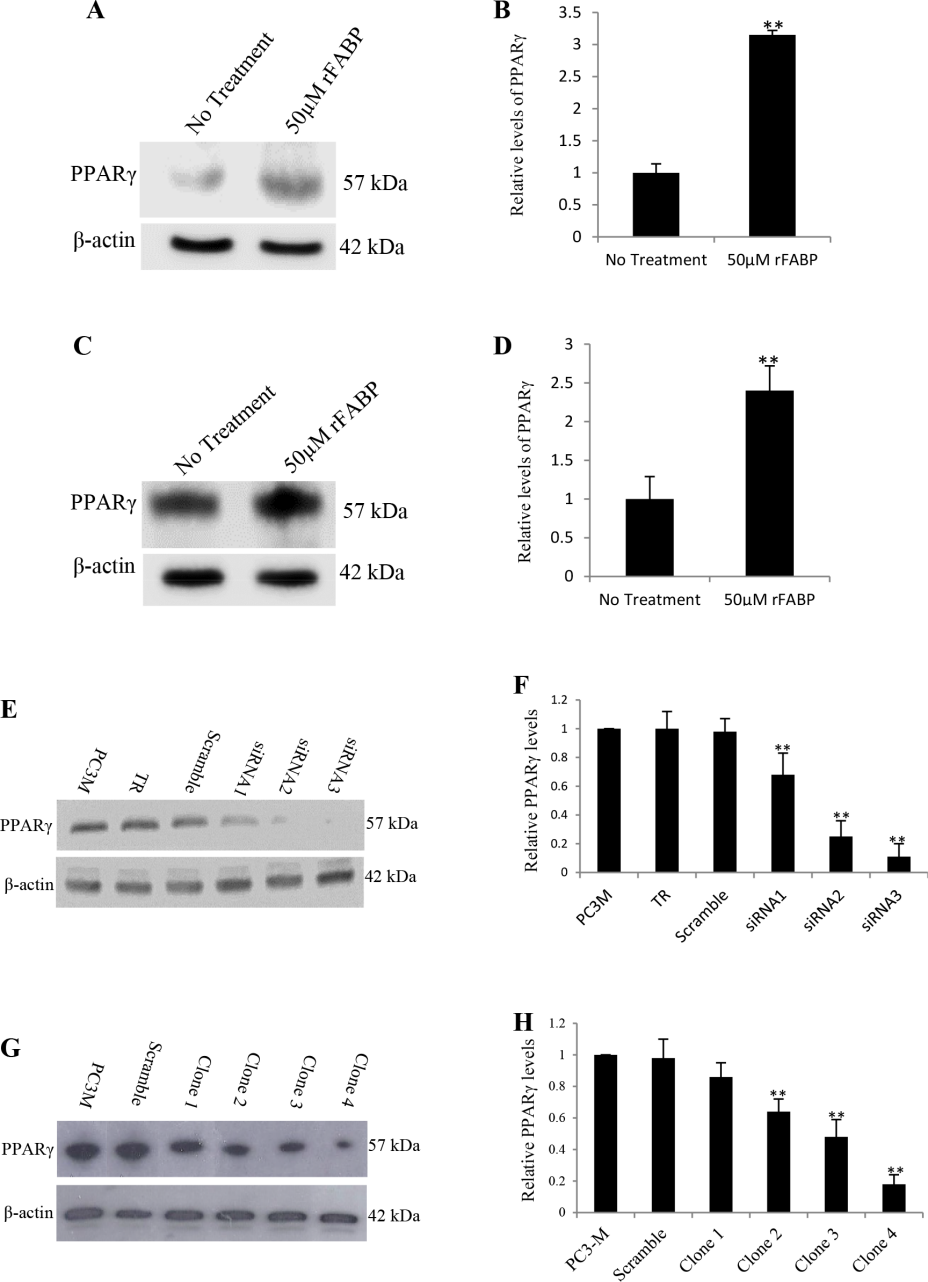
Increased expression of PPARγ by rFABP5 in LNCaP and 22RV1 cells and establishment of colonies expressing reduced level of PPARγ by siRNA in PC3-M cells Quantitative analysis of the levels of PPARγ in different cells was performed by densitometry scan of the intensities of the bands of the Western blots. Quantitative results were obtained from 3 repeated blot analyses. **A.** Western blot of PPARγ in untreated LNCaP cells and in cells treated with 50μM rFABP5 at presence of 2μM myristic, palmitic, oleic and linoleic acids for 24h. **B.** Quantitative analysis of PPARγ in control and in rFABP5 treated (at the presence of long chain fatty acids) LNCaP cells. The level of PPARγ in untreated control cells was set at 1; and the level in rFABP5 treated cells was obtained by relating that to the control. **C.** Western blot of PPARγ in untreated 22RV1 cells and in cells treated with 50μM rFABP5 at presence of 2μM myristic, palmitic, oleic and linoleic acids for 24h. **D.** Quantitative analysis of PPARγ in control and in rFABP5 treated (at the presence of long chain fatty acids) 22RV1 cells. The level of PPARγ in untreated control cells was set at 1; and the level in rFABP5 treated cells was obtained by relating that to the control. **E.** Western blot of PPARγ in parental PC3-M cells, cells treated with transfection reagent (TR) only, or cells transfected transiently with scrambled RNA and 3 different siRNA molecules. To standardize the immuno-blot reaction, an anti-β-actin was incubated with the same blot. **F.** Quantitative analysis of the levels of PPARγ in PC3-M cells after transient transfection. The level of PPARγ in parental PC3-M was set at 1; Levels in other transfected cells were obtained by comparison with that in PC3-M. **G.** Western blot analysis of the levels of PPARγ in parental PC3-M cells, cells transfected with scrambled shRNA and 4 different clones derived from siRNA-3 stable transfectants. An anti-β-actin was incubated with the same blot to normalize possible loading errors. **H.** Quantitative analysis of the levels of PPARγ in parental PC3-M cells, the scrambled shRNA control cells and 4 different clones derived from RNAi-3 stable transfectants. The level of PPARγ in parental PC3-M was set at 1; Levels in other transfected cells were obtained by comparison with that in PC3-M.

To confirm the effect of FABP5 on PPARγ, wild type recombinant FABP5 (rFABP5) was used to stimulate prostate cancer cells. Western blot analysis (Fig. 1A and Fig. 1C) showed that the rFABP5 stimulation produced 3.15±0.7 fold increase in PPARγ expression in LNCaP cells (Fig. 1B) and 2.14±032 fold increase in 22RV1 cells (Fig. 1D). To identify the best PPARγ suppresser, PC3-M cells were transiently transfected for 24 hours with 3 candidate double-stranded siRNAs and the changes in PPARγ were measured by Western blot (Fig. 1E). When the expression level of PPARγ in parental PC3-M cells was set at 1.0, the relative levels in cells transfected with siRNA 1, 2 and 3 were 0.68 ± 0.15, 0.25 ± 0.11 and 0.11 ± 0.09, respectively (Fig. 1F), the most significant reduction (up to 89%) (Student's t-test, *p* < 0.001) was achieved by siRNA-3. Thus siRNA-3 was selected as the most efficient suppressing sequence to design shRNA for stable transfection. The shRNA sequence of siRNA-3 was cloned into the psiRNA-h7SKGFPzeo plasmid and stably transfected into PC3-M cells to knockdown PPARγ. Western blots of separate cell lines established from individual colonies of transfectants showed a single PPARγ band of 57 kDa (Fig. 1G). When the level of PPARγ in the parental PC3-M cells was set at 1, the level in scrambled RNA control cells was 0.98 ± 0.11 and in clones 1-4 was 0.86 ± 0.09, 0.64 ± 0.08, 0.48 ± 0.11 and 0.18 ± 0.06, respectively (Fig. 1H). Thus levels of PPARγ were significantly suppressed by 14-52% (Student's t-test, *p* = 0.007) in colonies 1-3 and by 82% (Student's t-test, *p* = 0.0008) in clone 4 cells; there was no significant change in scrambled RNA control transfectants. Thus cell lines established from clone 3 and clone 4 were selected as moderately and highly *PPARγ*-suppressed transfectants and termed PC3-M-PPARγ-si-M and PC3-M-PPARγ-si-H, respectively.

### Effect of *PPARγ* suppression on malignant properties *in vitro* and tumorigenicity *in vivo*

**Figure 2:**
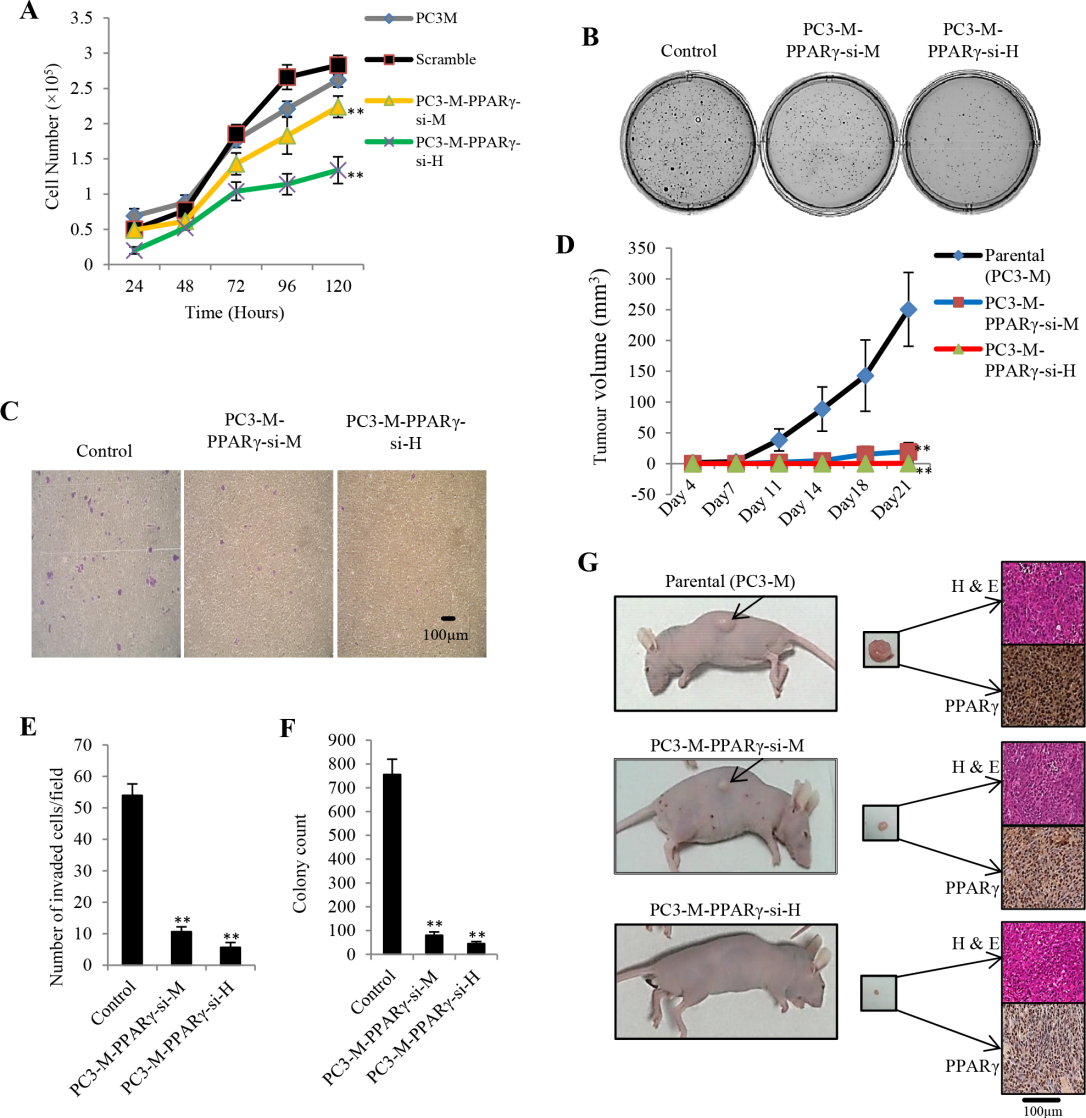
Effect of PPARγ suppression on malignant characteristics of PC3-M cells *in vitro* and their tumourigenicity in *vivo* **A.** Effect of PPARγ suppression on proliferation of the transfectant cells over the 5 day experimental period. Results were obtained from 3 separate experiments (mean ± SE). **B.** Effect of PPARγ suppression on anchorage-independent growth of tranfectant cells: Representative plates of cell colonies growing in soft agar with different transfectants. **C.** Effect of PPARγ suppression on invasiveness of transfectant cells: Representative Fields of invasion assays are shown. Original magnifications: 100×. **D.** Average volume of tumors produced by each group of nude mice 3 weeks after inoculation with transfectant cells. **E.** Number of invaded cells per field in invasion assay. Results are obtained from three separate measurements (mean ± SE). **F.** Colony counts of different transfectants growing in soft agar after 4 weeks. Results are obtained from three separate plates in the same experiment (mean ± SE). **G.** A representative mouse and its corresponding tumor mass from each group. Presence of tumor cells in all tumor masses has been confirmed by H&E staining (upper figure for each tumor). Immunohistochemical staining with PPARγ antibody showed remarkable differences in PPARγ expression between test groups and the control (lower figure for each tumor). Original magnifications of slides: 250×.

Effects of *PPARγ*-suppression on proliferation rate, anchorage-independent growth and invasiveness of prostate cancer cells were evaluated by a proliferation assay (Fig. 2A), a soft agar assay (Fig. 2B) and an invasion assay (Fig. 2C), respectively. Overall, parental cells and control (scrambled) exhibited very similar growth patterns when tested with the MTT proliferation assay. In the first 48 hours, no significant difference was detected amongst all tested cell lines. But from the 3^rd^ day onward, cell numbers were significantly reduced in both PC3-M-PPARγ-si-H and PC3-M-PPARγ-si-M compared to controls. At the end of the 5^th^ day, cell numbers in PC3-M and scrambled RNA groups reached 262,000 ± 14,000 and 283,000 ± 9810, respectively; whereas in PC3-M-PPARγ-si-H cells and PC3-M-PPARγ-si-M cells, they were significantly reduced by 53% and 21% to 134,000 ± 19,040 and 224,000 ± 15,200, respectively (Student's t-test, *p* = 0.009 and *p* = 0.004). Mean number of invaded cells in scrambled (control), PC3-M-PPARγ-si-M and PC3-M-PPARγ-si-H group was 54 ± 4, 11 ± 2 and 6 ± 1, respectively (Fig. 2E); representing a significant reductions of 79.7% and 88.91%, respectively (Student's t-test, *p* = 0.0002, *p* = 0.0003). The number of colonies produced in soft agar after 4 weeks by control, PC3-M-PPARγ-si-M and PC3-M-PPARγ-si-H cells were 756 ± 64, 80 ± 14 and 45 ± 9, respectively (Fig. 2F), representing significant reductions of 89.5% and 94.1%, respectively (Student's t-test, *p* = 0.0015, *p* = 0.0012).

To evaluate the effect of *PPARγ* suppression on the tumorigenicity of PC3-M cells *in vivo*, parental and transfectant cells were injected subcutaneously into the flanks of nude mice. The median latent period for the group with PC3-M-PPARγ-si-H cells was 21 days, significantly longer than those with parental cells [6 days (range 5-14)] and with PC3-M-PPARγ-si-M cells [11 days (range 7-19)]. After three weeks, only 1 of 10 (10%) mice with PC3-M-PPARγ-si-H cells produced a visible tumour, whereas all mice (100%) with parental cells and 7 out of 10 (70%) with PC3-M-PPARγ-si-M cells produced tumours (Table 1A). At the end of this experiment, average volume of tumours produced by control cells (250.6 mm^3^ ± 60) was significantly larger than those produced by PC3-M-PPARγ-si-M cells (19.5 mm^3^ ± 14.6) (Student's t-test, *p* = 0.008) and PC3-M-PPARγ-si-H cells (2 mm^3^) (*p* = 0.0009) (Fig. 2D). At autopsy (three weeks after inoculation), average weight of the tumours produced by the parental cells was 275 mg ± 105 which was significantly higher than those produced by PC3-M-PPARγ-si-M cells (26.5 mg ± 12.6) (*p* = 0.002) and PC3-M-PPARγ-si-H cells (4 mg) (*p* = 0.0003). Representative nude mice from each group, corresponding tumour mass, H&E and immunohistochemical staining of tumour tissues are shown in Fig. 2G. In control group, 5 (50%) sections stained moderately positive and another 5 (50%) sections stained strongly positive in the nucleus (Table 1B). Among a total of 7 stained sections from PC3-M-PPARγ-si-M group, 2 (29%) stained weakly and 5 (71%) stained moderately positive in the nucleus. Nuclear expression of PPARγ in the only tumour produced by PC3-M-PPARγ-si-H cells was weakly positive. No significant difference in cytoplasmic expression of PPARγ in different primary tumours was detected. Intensity of staining for PPARγ in tumours produced by PC3-M-PPARγ-si-H cells (Fisher Exact test, *p* = 0.002) or PC3-M-PPARγ-si-M cells (*p* = 0.02) was significantly weaker compare to those produced by parental cells.

Table 1Tumors produced in nude mice and expression of PPARγ in tumor cells

**A:** Incidence, latent period and average tumor weight

Cell lines inoculated	No. of mice	Incidence of tumors ^*I*^		Median of latent period (range) ^*II*^	Average tumor weight (mg) ^*III*^
		No.	%		
PC3-M-PPARγ-si-H	10	1	10	21	4
PC3-M-PPARγ-si-M	10	7	70	11 (7-19)	26.5 ± 12.6
PC3-M (control)	10	10	100	6 (5-14)	275 ± 105

I Tumor incidence is number of mice developing tumors and percentage is the incidence divided by total number of tested mice.

II Latent period is the number of days from the inoculation time to the time of first appearance of tumor.

III Tumor weight was measured at autopsy on 21^st^ day after inoculation of the transfectant cells.

**B:** Nuclear expression of PPARγ in cells of tumors resected from nude mice

Origin of tissue (mice groups)	No. of cases	Nuclear staining score (1-9)		
		≤ 3	4-6	≥ 9
Control	10	0	5	5
PC3-M-PPARγ-si-M	7	2	5	0
PC3-M-PPARγ-si-H	1	1	0	0

### FABP5 and PPARγ up-regulated *VEGF* expression

**Figure 3:**
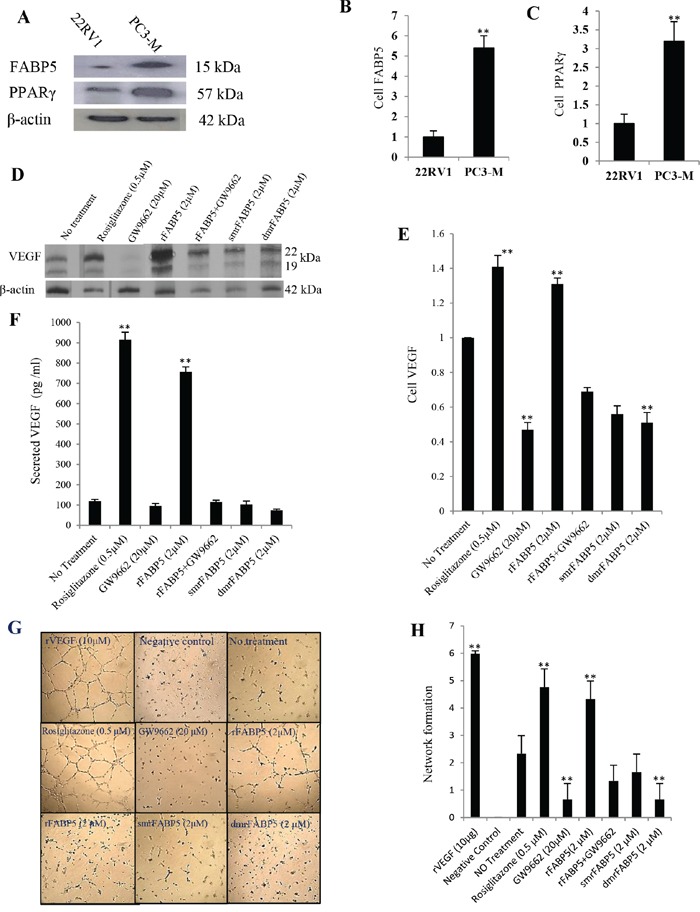
Up-regulation of VEGF by FABP5 or PPARγ and its biological activity in prostate cancer cells. Significantly different results between control and test groups were marked with double asterisks **A.** Western blot of FABP5 and PPARγ in androgen-dependent 22RV1 and androgen-independent PC3-M cells. The anti-β-actin antibody was used to correct possible loading errors in the same blot. **B.** Relative expression of FABP5. **C.** Relative expression of PPARγ. The levels of both proteins in 22RV1 cells were set as 1.0 and levels in PC3-M cells were calculated by relating to those in 22RV1. Results were obtained from three different experiments (mean ± SE). From **D to H.** levels of VEGF produced by androgen-dependent 22RV1 cells and its biological activity after cells were treated overnight in different conditions. **D.** Western blot analysis of VEGF protein in 22RV1cells before and after different treatments (antibody reacts with two VEGF splice variants with molecular weights of 19 & 22 kDa). The anti-β-actin was used to correct possible loading errors in the same blot. **E.** Relative levels of VEGF. Level of VEGF in untreated 22RV1 cells was set at 1.0; levels in other cells were calculated by relating to that in untreated 22RV1 cells. Results were obtained from three different experiments (mean ± SE). **F.** ELISA measurement of levels of secreted VEGF in 22RV1 conditioned media obtained after different treatments. Results were obtained from three different experiments (mean ± SD). **G.** HUVEC cells’ network formation on ECMatrix, exposed to different 22RV1 conditioned media. Original magnifications of representative slides: 250×. The positive control is human rVEGF (10μg/ml) and the negative control is the normal cell culture medium. **H.** Relative values of HUVEC cells’ network formed by addition of different 22RV1 conditioned media. Three individual assays were performed for each treatment and the values (mean ± SD) were obtained from five random fields in each assay.

Moderately malignant 22RV1 cells were used to study the effect of PPARγ and FABP5 on VEGF expression. Quantitative analysis showed that levels of FABP5 and PPARγ in PC3-M were respectively 5.4- and 3.2- fold higher than those in 22RV1 (Fig. 3A, 3B, 3C). To test the role of FABP5 on up-regulation of VEGF (through PPARγ), 22RV1 cells were treated with PPARγ synthetic agonist (rosiglitazone, 0.5 μM) or recombinant (r) FABP5 protein (2 μM) overnight. Western blot analysis on protein extracts detected two VEGF bands of 19 and 22kDa (representing VEGF_121_ and VEGF_165_ isoforms, respectively) in both untreated and treated cells (Fig. 3D). When the densitometry measurement level of VEGF in untreated 22RV1 cells was set at 1, VEGF levels in cells treated with rosiglitazone and rFABP5 were 1.41 ± 0.12 and 1.36 ± 0.08 (Fig. 3E); significant increment of 41% and 36% (Student t-test, *p* = 0.006), respectively. The increment was based on the intensities of both bands. Levels of secreted VEGF in conditioned media were assessed by ELISA (Fig. 3F). The amount of VEGF secreted by 22RV1 cells without any treatment was 120 ± 8.3 pg/ml, while those treated with rosiglitazone (0.5 μM) or rFABP5 (2 μM) were significantly increased to 915 ± 37.8 pg/ml (7.6 times; Student t-test, *p* = 0.0003) and 756 ± 25.6 pg/ml (6.3 times; *p* = 0.0005), respectively. Angiogenesis *in vitro* of secreted VEGF from 22RV1 cells was evaluated by the ability to stimulate human umbilical vein endothelial cells (HUVEC) to form tubes (Fig. 3G). The tube-forming ability of HUVEC were strongly promoted and well-assembled organizations formed in those stimulated with media of rosiglitazone-treated cells or rFABP5, similar to a positive control with 10ng/ml recombinant human (rh)VEGF. Conditioned medium from the untreated 22RV1 cells caused partially visible sprouting of new capillary tubes. The average numerical values associated with tube formation showed a significant increase in cells treated with rosiglitazone (2-fold) (Student's t-test, *p* = 0.003) or rFABP5 (1.8-fold) (*p* = 0.006) compared to untreated cells.

To investigate whether FABP5 suppression can down-regulate VEGF expression through PPARγ, 22RV1 cells were treated overnight with PPARγ synthetic antagonist (GW9662) (20 μM) and 2 mutant FABP5 recombinant proteins (2 μM). These mutant proteins were generated by a single mutation (sm) or double mutations (dm) to change 1 or 2 of the 3 amino acids respectively in the fatty acid-binding motif of FABP5 [14]. While the fatty acid-binding capacity of smrFABP5 is partially lost, dmrFABP5 is almost incapable of binding to fatty acids. When the level of VEGF in untreated 22RV1 cells was set at 1, levels in those treated with GW9662, smrFABP5 and dmrFABP5 were 0.47 ± 0.08 (Student t-test, *p* = 0.005), 0.56 ± 0.08 (*p* = 0.007) and 0.49 ± 0.12 (*p* = 0.004), respectively (Fig. 3D, 3E). The amount of VEGF secreted by the cells treated with GW9662 (20 μM), smrFABP5 and dmrFABP5 (2 μM) were 96 ± 12.1 pg/ml, 102 ± 18.4 pg/ml and 74 ± 6.2 pg/ml; significant reductions were produced by GW9662 (20%; Student's t-test, *p* = 0.002), and dmrFABP5 (39%; *p* = 0.0009) respectively (Fig. 3F), but not by smrFABP5 (15%, *p* = 0.1). When tested for *in vitro* angiogenesis by treatment with GW9662 or dmrFABP5, HUVEC cells remained randomly separated without any sign of a formation of a complex mesh; similar to that of the negative control (cells treated with normal culture medium) (Fig. 3G). Conditioned medium of cells treated with smrFABP5 induced only some visible capillary tubes without any sprouting. The average numerical values associated with tube formation showed a prominent (72%) significant reduction in cells treated with GW9662 or dmrFABP5 in comparison to untreated 22RV1 cells (Student's t-test, *p* = 0.01), whereas that for the smrFABP5 was not (Student's t-test, *p* = 0.11). These results suggest that the fatty acid ligands of FABP5 is responsible for the increased production of biologically active VEGF in 22RV1 cells.

To study the possible effect of PPARγ inhibition on counteracting up-regulatory effects of FABP5 on VEGF, 22RV1 cells were treated with PPARγ antagonist GW9662 (20 μM), rFABP5 (2 μM), or a combination of rFABP5 with GW9662, overnight. When the level of VEGF in untreated 22RV1 cells was set at 1, levels in those treated with GW9662, rFABP5 and mixture of rFABP5 with GW9662 were 0.47 ± 0.08, 1.36 ± 0.08 and 0.69 ± 0.04, respectively (Fig. 3E). Although level of VEGF showed a 36% increase after treating with rFABP5, a 31% decrease was detected after treating with a mixture of rFABP5 and GW9662. The VEGF level in GW9662 treated cells is significantly lower than that in cells treated by GW9662 and rFABP5 jointly (Student t-test, *p* = 0.02). Levels of secreted VEGF in conditioned media (Fig. 3F) produced by untreated 22RV1 cells, by cells treated with GW9662 (20 μM), and by rFABP5 alone or a mixture of rFABP5 with GW9662 were 120 ± 8.3, 96 ± 12.1, 756 ± 25.6 and 105 ± 9.6 pg/ml, respectively. Although treatment with rFABP5 alone produced a 6.3-fold increase in secreted VEGF, when the rFABP5 was combined with GW9662, the level of secreted VEGF was significantly reduced (Student t-test, *p* = 0.0005) to 12.5% below the control. Moreover, the average numerical values associated with tube formation showed a significant increase (*p* = 0.006) in the cells treated with rFABP5 (1.8-fold); whereas in those cells treated with combination of rFABP5 and GW9662, a 42% reduction (Fig. 3H) was observed, although it was not significantly different from their control (Student's t-test, *p* = 0.057).

### *PPARγ* regulates *VEGF* expression through acting with PPREs in *VEGF* promoter

**Figure 4:**
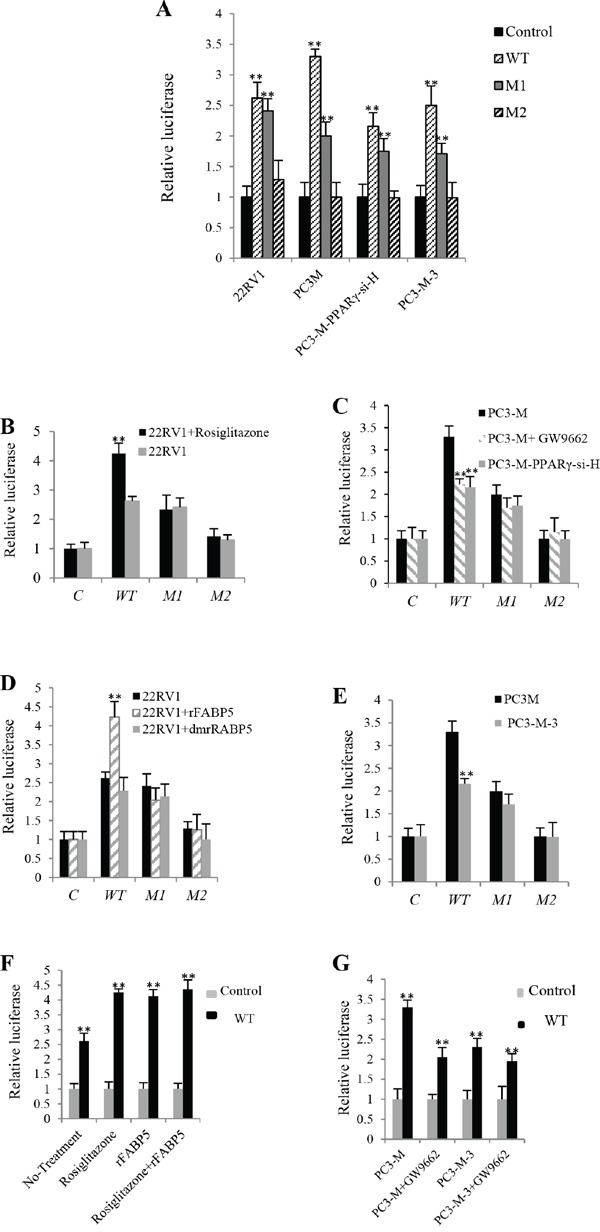
The effect of PPREs on levels of Luciferase activities in prostate cancer cells transfected with different VEGF gene reporter vectors Following 4 vectors were used to transfect the different prostate cancer cell lines: The control plasmid (pGL3-promoter-only), WT, M1 and M2. Luciferase activity of the cells transfected with control vector was set at 1; levels in other cells were calculated by relating to that of the control. Results were obtained from three different experiments (mean ± SD). *Renilla* luciferase plasmid was co-transfected into cells in every transfection as an internal control to minimize experimental viability caused by possible differences of cell viability or transfection efficiency. **A.** Relative luciferase activities in 22RV1, PC3-M, PC3-M-PPARγ-si-H and PC3-M-3 cells transfected with VEGF-promoter-reporter constructs. **B.** Relative luciferase activities in transfected 22RV1 cells before and after treatment with the PPARγ agonist (Rosiglitazone, 0.5μM). **C.** Relative luciferase activities in transfected PC3-M cells before and after treatment with the PPARγ antagonist (GW9662, 20μM) and those in transfected PC3-M-PPARγ-si-H cells without any treatment. **D.** Relative luciferase activities in transfected 22RV1 cells before and after treatment with rFABP5 (2μM) or dmrFABP5 (2μM). **E.** Relative luciferase activities in transfected PC3-M and PC3-M-3 cells without any treatment. **F.** Relative luciferase activities in 22RV1 transfectant cells (transfected with WT or control plasmid) before and after treating with rosiglitazone (0.5μM) and rFABP5 (2μM) either individually or jointly. **G.** Relative luciferase activities in PC3-M and PC3-M-3 transfectant cells before and after GW9662 (20μM) treatment.

To investigate whether the activated PPARγ (by fatty acids transported via FABP5) up-regulated VEGF by binding to *PPRE*s in the promoter region of the *VEGF* gene, 22RV1 cells, PC3-M cells, PC3-M-3 cells (*FABP5*-suppressed PC3-M cells) and PC3-M-PPARγ-si-H cells (*PPARγ*-suppressed PC3-M cells) were co-transfected with following 4 reporter constructs: Control plasmid (pGL3-promoter-only), Wild type (WT) which contains PPREs, Mutant 1 (M1) which contains the same segment of DNA as WT except that both PPREs are mutated, and Mutant 2 (M2) which contains a much shorter DNA segment without PPREs. All plasmids, the control, WT, M1 or M2, contain the luciferase coupled promoters of VEGF gene. When the level of luciferase activity in 22RV1 cells transfected with control plasmid was set as 1 (Fig. 4A), levels in those transfected with WT, M1 and M2 promoters were 2.62 ± 0.26, 2.41 ± 0.25 and 1.29 ± 0.17, respectively. When the level of luciferase activity in PC3-M cells transfected with control plasmid was set as 1, levels in those transfected with WT, M1 and M2 promoters were 3.3 ± 0.12, 2 ± 0.14 and 1 ± 0.21, respectively. When the level of luciferase activity in PC3-M-PPARγ-si-H cells transfected with control plasmid was set as 1, levels in those transfected with WT, M1 and M2 plasmids were 2.16 ± 0.22, 1.75 ± 0.24 and 0.99 ± 0.23, respectively. When the level of luciferase activity in PC3-M-3 cells transfected with control plasmid was set as 1, levels in those transfected with WT, M1 and M2 promoters were 2.5 ± 0.32, 1.71 ± 0.31 and 0.99 ± 0.2, respectively. In this set of transfections, only WT and M1 produced significant increment in luciferase activity.

To investigate further the effect of PPARγ on *VEGF*-promoter activity, 22RV1 cells were transfected with different reporter promoters and treated with the PPARγ agonist rosiglitazone (0.5μM). Levels of luciferase activity in 22RV1 cells transfected with WT, M1 and M2 rose to 4.25 ± 0.35, 2.33 ± 0.50 and 1.42 ± 0.26, respectively (Fig. 4B). Compared to the untreated control, the luciferase activity in cells transfected with WT (Student's t-test, *p* = 0.0003) and not mutant promoters (*p* ≥ 0.25) was significantly higher. After treating with the PPARγ antagonist GW9662 (20μM), levels of luciferase activity in PC3-M cells transfected with WT, M1 and M2 were reduced to 2.23 ± 0.12, 1.70 ± 0.22 and 1.15 ± 0.32, respectively over the untreated control, which were similar to the levels obtained in co-transfected PC3-M-PPARγ-si-H cells (Fig. 4C). Only that for WT (Student's t-test, *p* = 0.002) was significantly lower; those for M1 (*p* = 0.08) and M2 (*p* = 0.27) were not. No significant difference was detected between levels of luciferase in transfected PC3-M cells treated with GW9662 and that in PC3-M-PPARγ-si-H cells (*p* ≥ 0.22).

To study the effect of FABP5 on *VEGF*-promoter activity, cells were treated with rFABP5. Levels of luciferase activity in 22RV1 cells co-transfected with WT, M1 and M2 were increased to 4.23 ± 0.41, 2.04 ± 0.32 and 1.26 ± 0.40, respectively. After treating with dmrFABP5, luciferase activities in 22RV1 cells transfected with WT, M1 and M2 were 2.29 ± 0.35, 2.14 ± 0.32 and 1.1 ± 0.41, respectively (Fig. 4D). That for WT (Student's t-test, *p* = 0.004) was significantly higher than the control; those for mutant promoters were not (Student's t-test, *p* = 0.44, *p* = 0.09). No significant difference was detected in luciferase activities between transfected 22RV1 cells treated with dmrFABP5 and the cells in the control (*p* = 0.33). Comparing with PC3-M cells, the luciferase level in PC3-M-3 cells transfected with WT (Student's t-test, *p* = 0.001) was significantly lower; whereas those transfected with either M1 or M2 (Student's t-test, *p* ≥ 0.07) was not significantly different from the control (Fig. 4E).

To study the combined effects of FABP5 and *PPARγ* on *VEGF*-promoter activity, the luciferase activity of 22RV1 cells transfected with control plasmid was set as 1, the activity of those transfected with WT and treated with rosiglitazone (0.5μM), rFABP5 (2μM) and rosiglitazone plus rFABP5 were 4.25 ± 0.12, 4.13 ± 0.22 and 4.36 ± 0.32, respectively. Activity in untreated 22RV1 cells was 2.62 ± 0.26 (Fig. 4F). The luciferase activities in cells transfected with WT and treated with either rosiglitazone (Student's t-test, *p* = 0.0003), rFABP5 (Student's t-test, *p* = 0.004) or combination of rosiglitazone and rFABP5 (Student's t-test, *p* = 0.001) were significantly higher in comparison to untreated control cells; whereas they were not significantly different when compared to each other (*p* = 0.17). When the level of luciferase activity of PC3-M cells transfected with control plasmid was set as 1, activity in cells transfected with WT was 3.3 ± 0.18. Activities in PC3-M cells treated with GW9662 (20μM), untreated PC3-M-3 cells and PC3-M-3 cells treated with GW9662 (20μM) were 2.05 ± 0.24, 2.23 ± 0.21, 1.95 ± 0.19, respectively (Fig. 4G), all significantly lower (*p* ≤ 0.001), whereas these levels were not significantly different when compared to each other (*p* ≥ 0.25).

### Effects of Sp1 (androgen binding site) on *VEGF*-promoter activity

**Figure 5:**
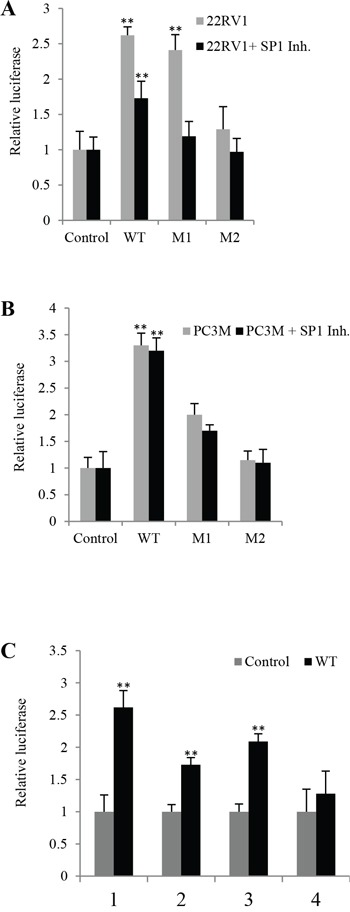
The effect of SP1 inhibitor on levels of luciferase activities in prostate cancer cells transfected with different VEGF gene reporter vectors Luciferase activity of the cells transfected with control was set as 1; levels in other cells were calculated by relating to that of the control. Results were obtained from three different experiments (mean ± SD). *Renilla* luciferase plasmid was co-transfected into cells in every transfection as an internal control to minimize experimental variability caused by difference of cell viability or transfection efficiency. **A.** Relative luciferase activities in 22RV1 transfectants before and after Sp1 inhibitor (Mithramycin A, 0.1μM) treatment. **B.** Relative luciferase activities in PC3-M transfectants before and after Sp1 inhibitor treatment. **C.** Combined effects of Sp1 and *PPARγ* on *VEGF*-promoter activity in prostate cancer cells. Relative luciferase activities in 22RV1 transfectants (22RV1 cells transfected with control and WT) before and after different treatments; (1) Untreated 22RV1 transfectants, (2) 22RV1 transfectants treated with Sp1 inhibitor mithramycin A, (3) 22RV1 transfectants treated with PPARγ antagonist GW9662, (4) 22RV1 transfectants treated with both mithramycin A and GW9662.

To study the possible effect of the androgen binding site on VEGF-promoter activity, cells were first treated with the Sp1 inhibitor mithramycin A (0.1μM) and then transfected with different DNA reporter constructs. When the luciferase activity in the cells transfected with the control was set at 1, luciferase activities in androgen-sensitive 22RV1 cells transfected with WT, M1 and M2 were 1.73 ± 0.11, 1.19 ± 0.24 and 0.97 ± 0.31, respectively (Fig. 5A). Thus, after treatment with mithramycin A the luciferase activity in cells transfected with WT (Student's t-test, *p* = 0.007) and M1 promoters were significantly lower (Student's t-test *p* = 0.001) than the untreated conrol; but not in cells transfected with M2 (*p* ≥ 0.1). After treating with mithramycin A, luciferase activities in androgen-independent PC3-M cells transfected with WT, M1 and M2 promoters were 3.2 ± 0.23, 1.7 ± 0.11, 0.95 ± 0.25, respectively (Fig. 5B), none were significantly lower than the untreated control (*p* ≥ 0.06).

When luciferase activity in 22RV1 cells transfected with control plasmid (pGL3-promoter) was set as 1, activities in those transfected with WT but without any other treatment was 2.62 ± 0.26; activities in those cells transfected with WT plasmid and treated with Sp1 inhibitor mithramycin A (0.1μM), PPARγ antagonist GW9662 (20μM) and combination of mithramycin A with GW9662 were 1.73 ± 0.11, 2.09 ± 0.12 and 1.28 ± 0.35, respectively (Fig. 5C). Thus the luciferase activity in cells transfected with WT and treated with mithramycin A and GW9662 was significantly lower (Students t-test, *p* = 0.026) but not between cells transfected with control plasmid and those transfected with WT and treated with combinations of mithramycin A and GW9662 (*p* = 0.085).

## DISCUSSION

### Suppression of *PPARγ* reduced the tumorigenicity of prostate cancer cells

When assessing the role of PPARγ in tumorigenicity, siRNA knockdown rather than antagonist PPARγ was used to avoid any nonspecific effects [21]. When tested in 3 bioassays that are regarded as important criteria to measure tumorigenesis and capability of metastasis of cancer cells in *in vitro* [22], we showed (Fig. 2) that high levels of suppression of *PPARγ* in PC3-M cells (PC3-M-PPARγ-si-H) produced significant reductions in cell proliferation rate, invasiveness, and anchorage-independent growth. In nude mouse assay, suppression of *PPARγ* in PC3-M cells significantly reduced the size of tumours by 99%, tumour incidence by 90%. When the level of PPARγ was moderately suppressed in PC3-M cells, their malignant characteristics *in vitro* and their tumorigenicity in nude mice were inhibited, but to a limited degree. Thus the malignant characteristics are closely related to the level of PPARγ and that *PPARγ* played an important role in tumorigenicity of the prostate cancer cells. Previous studies showed that suppression of *FABP5* in PC3-M cells significantly reduced the same parameters [13, 23]. Thus combining current results with those of the past suggested that FABP5 and PPARγ may be functioning in a coordinated manner to promote the malignant progression of human prostate cancer cells.

### FABP5 promotes VEGF through PPARγ in prostate cancer cells

Increased level of PPARγ in carcinomas can stimulate angiogenesis through up-regulation VEGF or other pro-angiogenic factors [24-27]. Increased expression of *FABP5* induced metastasis through up-regulation of *VEGF* [12]; while suppression of *FABP5* inhibited tumorigenicity by decreasing VEGF expression [23]. Here, when 22RV1 cells were treated with rosiglitazone and rFABP5, both the cellular and secreted VEGF was remarkably increased and which caused great increases in angiogenic activity of the conditioned media. Conversely, when 22RV1 cells were treated with GW9662 and dmrFABP5, VEGF was greatly reduced in both cell extracts and conditioned media and the angiogenesis activity was greatly reduced. When the fatty acid-binding motif in FABP5 was attenuated by mutating 2 of the 3 key amino acids, it lost the ability of binding or transporting fatty acids [14]. Here when the cells were stimulated with dmrFABP5; there was a reduction in the level of VEGF and angiogenesis. These results suggest that the up-regulation of VEGF was produced by the increased cellular uptake of fatty acids transported by FABP5. When the ability of binding to and transporting fatty acids was lost, dmrFABP5 was not only incapable of inducing VEGF, it may also competitively inter-react with the fatty acid receptor to prevent FABP5 delivering fatty acids to their nuclear receptors and thus cannot initiate the down-stream cancer-promoting gene activity. This result suggested that dmrFABP5 can act as a suppressor to the tumorigenicity-promoting activity of FABP5 and PPARγ.

Like FABP5, PPARγ agonists can promote the up-regulation of VEGF and increase angiogenesis, whereas PPARγ antagonists can reduce VEGF and suppress angiogenesis. The suppression of tumorigenicity of prostate cancer cells by siRNA knocking down of PPARγ was likely to be achieved through inhibiting the biological activity of VEGF. In contrast, the result that FABP5 plus PPARγ antagonists could not induce up-regulation of VEGF expression suggested that FABP5 promoted VEGF expression and angiogenesis via PPARγ (through the stimulation of the fatty acids transported by FABP5). When PPARγ was blocked with its antagonists, it did not respond to the stimulation signal produced by fatty acids, even when high level of fatty acids was available.

### PPARγ upregulated *VEGF* expression acts via the *PPRE*s in the *VEGF* promoter

Although androgen can mediate the upregulation of VEGF expression in androgen-dependent prostate cancer cells through the Sp1/Sp3 binding site in the *VEGF* core promoter [28], it was not previously known how PPARγ exactly up-regulated *VEGF* in prostate cancer cells. Studies showed that the regulatory effect of PPARγ ligands on *VEGF* expression in human endometrial cells was modulated through *PPRE*s in the promoter region of the *VEGF* gene [29]. To investigate whether activated PPARγ upregulates *VEGF* and promotes angiogenesis in prostate cancer cells in a similar way, a luciferase reporter promoter system was employed. Although the promoter region of the *VEGF* gene is relatively long and contains many sequences of *PPRE*s, the efficiency in promoting the reporter gene expression generated by the full length (2274bp) and that produced by a truncated (790bp) segment of the *VEGF* promoter region were very similar [29]. Thus in this study, a truncated DNA segment containing 2 *PPRE*s, rather than the full-length promoter region was used to assess whether PPARγ up-regulates *VEGF* gene through *PPREs* in the promoter region.

When WT and the control plasmids were co-transfected into the 22RV1 cells, which expressed low levels of PPARγ and FABP5, and treated with the PPARγ agonist rosiglitazone, luciferase activity was significantly increased from 2.62 to 4.25; whereas in cells transfected with other constructs which did not contain *PPRE*s, the luciferase activity was not remarkably changed (Fig. 4B). Similarly, when the co-transfected highly malignant PC3-M cells, which expressed high levels of both PPARγ and FABP5, were treated with a PPARγ antagonist GW9662, the luciferase activity was remarkably reduced to that in M1 transfectants or in PPARγ-suppressed cells (PC3-M-PPARγ-si-H) (Fig. 4C). These results showed that the increased level of PPARγ and the presence of PPREs are essential for VEGF expression in prostate cancer cells and suggested that it was the interaction between PPARγ and the *PPRE*s in the *VEGF* promoter region that upregulated the VEGF expression.

When the co-transfected 22RV1 cells (with control plasmid and WT, M1 and M2, respectively) are treated with rFABP5, the level of luciferase activity of the WT promoter transfectants is increased by 62%, and no increase was observed in either M1 or M2 transfectants (Fig. 4D). This result suggests that FABP5 can promote VEGF expression only in the presence of *PPRE*s. When the same transfectants are treated with the mutant FABP5 which is incapable of binding fatty acids, no increment is observed in any of the 3 transfectants. This result suggested that it is the fatty acids transported by FABP5 that activate PPARγ and upregulate *VEGF* through *PPRE*s. This is further confirmed by the result that when expression of FABP5 is knocked down (PC3-M-3 cells), the luciferase activity is reduced by 35% compared to the co-transfected PC3-M cells (Fig. 4E).

**Figure 6:**
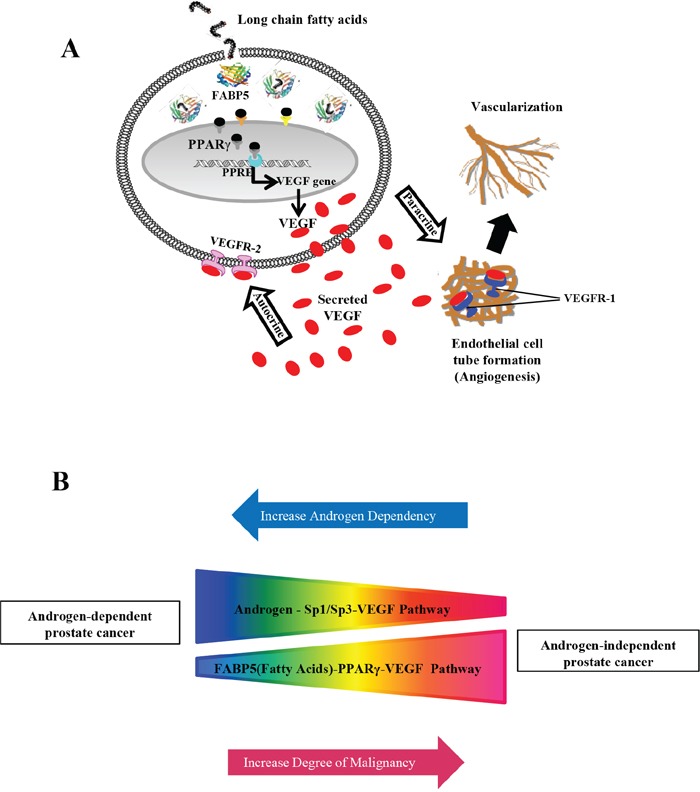
Schematic illustrations of how up-regulated VEGF and its biological activity promote tumorigenicity and how VEGF is upregulated through 2 different routes in prostate cancer cells **A.** Schematic illustration of “FABP5 (fatty acids)-PPARγ-VEGF” transduction axis. Through this pathway, fatty acids transported by FABP5 can activate PPARγ which ultimately upregulate VEGF. VEGF is a potent antigenic factor which can bind to its receptor VEGFR1 to promote formation of vessel networks that are essential for growth and expansion of the cancer cells. VEGF can also promote directly malignancy of the cancer cells through an autocrine mechanism to simulate the receptor that is highly expressed on the surface of the prostate cancer cells (VEGFR-2) (Gonzalez-Moreno et al, 2010). **B.** Schematic illustration of inter-relationship between androgen-Sp1/Sp3 (sugars) and FABP5 (fatty acids)- PPARγ signaling routes in up-regulating VEGF in androgen-dependent and androgen-independent prostate cancer cells. The results in this study suggested that in the early stage of prostate cancer, when the cancer cells are still responsive to androgen stimulation, the androgen-Sp1 pathway plays a dominant role in promoting VEGF expression. As the cancer cells gradually reduce their dependency on androgen supply and ultimately lose their responsive to androgen, the role of androgen-Sp1 pathway is gradually reduced and ultimately disappeared in AR-negative or androgen independent cancer cells. Opposite to this androgen-Sp1 pathway, the activity of FABP5-PPARγ-VEGF pathway in low malignant and androgen dependent cells (with low level of FABP5) is very low. But its activity is increased steadily as the increasing level of FABP5 and the malignancy of the cells and becomes dominant in CRPC cells.

After treating 22RV1 co-transfectants (transfected with WT) with PPARγ agonist (rosiglitazone) and rFABP5, the luciferase activity was increased by 62% and 57%, respectively; while subjecting them to a combination of both treatments can only raise the luciferase activity to a similar level of 66% (Student's t-test, *p* ≥ 0.17) (Fig. 4F). This result suggested that in 22RV1 cells expressing low levels of PPARγ and FABP5, both rosiglaitazone and rFABP5 can effectively increase VEGF expression by the same pathway. In contrast, luciferase activities in PC3-M co-transfectants (transfected with WT) treated with PPARγ antagonist (GW9662), the untreated PC3-M-PPARγ-si-H co-transfectants (transfected with WT) and PC3-M-3 co-transfectants (transfected with WT) are reduced by 32.5%, 35% and 34%, respectively (Fig. 4G). This result suggests that in PC3-M transfectant cells, which expressed high levels of both PPARγ and FABP5, suppressing the biological activity of PPARγ by either its antagonist or knocking down its mRNA by RNAi (as seen in PC3-M-PPARγ-si-H cells) can reduce the level of *VEGF* expression. Furthermore, suppression of *FABP5* expression in PC3-M cells can also reduce the level of VEGF (as seen in PC3-M-3). The difference between the level of luciferase activity in the untreated *FABP5*-knockdown PC3-M-3 cells and that in the cells treated with PPARγ antagonist is not significantly (Student's t-test, *p* = 0.25). This result indicates that when VEGF was already reduced by suppressing FABP5, little further reduction can be achieved by further treatment with PPARγ antagonist. These results taken together confirm both FABP5 and PPARγ are involved in an identical signalling pathway which regulates VEGF promoter activity in prostate cancer cells, as proposed schematically in Fig. 6A.

### Androgen- and FABP5-PPARγ-pathways for modulating *VEGF* expression and their relationship with androgen-dependency of the cancer cells

When 22RV1 cells are co-transfected with different luciferase constructs, the increase in VEGF expression produced by WT was similar to that produced by M1 which does not contain PPRE. Thus these increases cannot have been produced via the *PPREs,* but rather there are some other elements in the promoter region of the *VEGF* which are involved in up-regulating *VEGF* expression in 22RV1 cells. When PC3-M, PC3-M-PPARγ-si-H and PC3-M-3 cells were transfected with different constructs, both WT and M1 produced increases in luciferase activity. This result further confirmed that there are some other elements rather than PPREs that can stimulate VEGF expression in the highly malignant PC3-M cells (Fig. 4A).

Another possible important mechanism involved is the Sp1/Sp3 binding sites and their regulatory effect on VEGF activity has been shown in some other cancers, such as the up-regulation of VEGF by oestrogen in breast cancer cells via the Sp1/Sp3 transcription sites in the core VEGF promoter [31]. Similarly, androgen may mediate VEGF up-regulation in ADPC cells via Sp1/Sp3 binding sites [28]. In this work, after the 22RV1 cells were co-transfected with WT plus GW9662 and mithramycin A, luciferase activity was significantly reduced by 21% and 34%, respectively (Fig. 5C). These results indicate that in androgen responsive 22RV1 cells, both the FABP5-PPARγ and the androgen-Sp1 pathways may play important roles in up-regulating VEGF expression, although the androgen-Sp1 route appeared to be more active than the FABP5-PPARγ route. In contrast, when PC3-M co-transfectants (with WT) are treated with Sp1 inhibitor, luciferase activity was hardly reduced (Fig. 5B), whilst a significant reduction is achieved by GW9662 (Fig. 4C). These results suggest that in castration resistant PC3-M cells, the androgen-Sp1 pathway is no longer active in up-regulating VEGF and the extremely high level of VEGF is caused mainly through the FABP5-PPARγ route. This can be illustrated in Fig. 6B.

The androgen receptor (AR) plays a key role in prostate cancer carcinogenesis [32-34] and prostate cancer cells are generally sensitive to the initial androgen blockade treatment. But the cancer relapses afterwards with a more aggressive form, named castration-resistant prostate cancer (CRPC). CRPC does not depend on androgen anymore, but continuation of the androgen blockade therapy was still widely used to suppress CRPC [35, 36]. The molecular pathology involved in how androgen-dependent cancer cells are transformed to CRPC cells is not fully understood. Currently several mechanisms have been hypothesized [20]; the most common one is the AR sensitivity amplification theory [37, 38] which proposed that when deprived of androgen supply in the initial round of therapy, some cells try to maximise their survival ability by increasing the sensitivity of AR to make use of the small amount of reduced androgen which has escaped from the blockade. Thus some surviving cells with an increased ability of using microquantities of androgen become dominant and CRPC [39]. Some studies also suggested that mutations in *AR* gene can increase sensitivity to androgen [40]. Although each current theory can explain certain aspects of this pathological process, no single theory can satisfactorily explain all aspects. For example, if the AR amplification theory is correct, then re-expression of AR in AR-negative and highly malignant cells should increase the malignancy, but studies showed that the forced re-expression of AR in PC3 actually reduced the malignancy of such cells [41].

Based on the results of this and other studies, the following alternative hypothesis is proposed to explain the molecular mechanism involved in the crucial transition of cancer cells from androgen-dependent to an androgen-independent state: When the cancer cells are deprived of androgen supply, most of them die due to starvation, but some of them may have survived under the heavy selection pressure by switching their reliance on sugar (or glucose) uptake (regulated by androgen) [42] to fatty acid uptake (transported by FABP5) as an alternative energy source (so-called CRPCs). Although these cells can still use androgen, and further androgen blockade will kill some more cells, it can also make some other cells become even more resistant to androgen deprivation and increasing fatty acid intake; eventually, completely relying on fatty acids as their energy source. The consequence of the increased demand of fatty acids and hence the high level of FABP5 during this process is that the FABP5-PPARγ-VEGF axis gradually increases its functional activity and eventually replaces the androgen-Sp1/Sp3-VEGF pathway to become the dominant route to promote further malignant progression. Consequentially, prostate cancer cells will ultimately become totally androgen-independent after their androgen supply is repeatedly blocked. This can explain why androgen blockage can lead to a therapeutic dead end [43]. Based on this alternative hypothesis, disrupting the FABP5-PPARγ-VEGF signalling axis and cutting off the alternative energy supply of the cancer cells, rather than blocking the last drop of androgen, should be the correct way to kill the AR-negative CRPC cells.

## MATERIALS AND METHODS

### Cell lines and culture conditions

The following prostate cell lines were used in this study: 22RV1 cells, a moderately malignant and androgen-dependent cell line expressing low levels of FABP5 and PPARγ [44]; PC3-M cells, highly malignant and expresses very high levels of FABP5 and PPAR [45]; PC3-M-3 cells, a PC3-M-derived cell line established by knocking down *FABP5* [15]; PC3-M-PPARγ-si-M and PC3-M-PPARγ-si-H cells, PC3-M-derived cell lines established by knocking down *PPARγ* gene. Cells were cultured and maintained in RPMI 1640 medium (Invitrogen, Paisley, UK) supplemented with 10% (v/v) FCS (Biosera, East Sussex, UK), penicillin (100 units/ml) and streptomycin (100μg/ml) (Invitrogen).

### RNA interference

Three pairs of specific PPARγ siRNA sequences, chosen by Whitehead siRNA selection program, were purchased from Ambion (Life technologies, USA):

Sequence 1, sense strand 5′: GCCCUUCACUAC UGUUGACUU; antisence strand 5′: GUCAACAG UAGUGAAGGGCUU. Sequence 2, sense strand 5′: GGCUUCAUGACAAGGGAGUUU; antisence strand 5′: ACUCCCUUGUCAUGAAGCCUU. Sequence 3, sense strand 5′: UAAAUGUCAGUACUGUCGGUU; antisence strand 5′: CCGACAGUACUGACAUUUAUU. A scrambled siRNA (Ambion, Inc., USA) was used as negative control. PC3-M cells were transfected transiently with the X-tremeGENE siRNA Transfection Reagent (Roche) and Western blot was performed to measure PPARγ level. The siRNA sequence which caused the greatest reduction in level of PPARγ was chosen for short hairpin RNA (shRNA) construction. Two shRNA inserts consisting of PPARγ and scrambled siRNAs were designed using siRNA Wizard™ Software (InvivoGen, USA) and cloned into the psiRNA-h7SKGFPzeo plasmid (InvivoGen, USA) separately. X-tremeGENE HP DNA Transfection Reagent (Roche, Germany) was used to transfect the PC3-M cells with the vector containing PPARγ shRNAs or that harbored scrambled RNA. Transfected cells were cultured in a selective medium containing Zeocin (100μg/ml) (Life Technologies) for 3-4 weeks until the cell colonies were visualized. Five single colonies were isolated by ring cloning to establish PPARγ-suppressed sublines. Scrambled RNA transfectants were mixed to form a control pool.

### Proliferation assay

Transfectant cells were seeded in a 96-well plate in triplicate at a density of 1.25×10^4^ cells/200μl medium/well and colormetrically measured every day for 5 days, as described previously [15].

### Invasion assay

The BD BioCoat™ Matrigel™ Invasion Chamber (BD Biosciences, USA) was used to assess the invasiveness of the transfectants, as described previously [14].

### Soft agar assay

Transfectants and PC3-M cells were harvested and seeded in 6-well plates with low-melting agarose gel (5000 cells/well). After 4 weeks incubation, colonies were stained with 0.5 ml MTT (5 mg/ml, Sigma-Aldrich) for 4 hours. Colonies larger than 150μm were counted using an Optronix Gel Count (Oxford Optronix, UK).

### Tumorigenicity assay

The tumorigenicity of transfectants was tested by inoculating different cells in to male Balb/c 6-8 week old nude mice (Charles River Laboratories, UK). Cells (1×10^6^) suspended in 200μl PBS were injected subcutaneously into the flank of each mouse. Sizes of tumours were measured twice a week using callipers. Tumour volumes were calculated by the L×W×H×0.5236 formula [46] and all tumours were weighed at autopsy (3 weeks after the inoculation). The work was conducted under UKCCCR guidelines (HO Licence PPL 40/3578).

### Immunohistochemical staining

Histological sections (4μm) were cut from formalin-fixed, paraffin-embedded primary tumors [47] that were removed by dissection, incubated at 37°C overnight, deparaffinised with xylene and stained with haematoxylin and eosin with an automated Varistain 24-4 machine (Thermo Scientific, USA). The antibodies against FABP5 (Hycult, Netherlands), PPARγ (Santa Cruz, USA) and VEGF (Thermo Scientific, USA) were purchased commercially and the procedures for immunohistochemical staining were similar to those used previously [20].

### Scoring immunoreactivity

Evaluation of PPARγ immunoreactivity was performed in high power fields (×400) using a standard light microscope. Nuclear immunoreactivities were independently reviewed by two separate observers. Nuclear staining was first assessed by the number of stained nuclei to obtain a percentage score which was 1 (≤30%), 2 (31-60%), and 3 (≥61%); then by the intensity of staining to obtain an intensity score which was 1 (+), 2 (++), or 3 (+++). The final score for nuclear staining was obtained by multiplying the percentage and intensity scores. The final nuclear stains, which scored from 1 to 9, were further classified into weakly positive (1-3), moderately positive (4-6) and strongly positive (7-9), as described previously [48].

### VEGF ELISA assay

22RV1 cells (1×10^6^ cells/well) were seeded in a 6 well plate and cultured in growth factor-deprived medium (phenol red-free RPMI 1640 containing 10% charcoal stripped FCS) for 48 hours then exposed to different treatments overnight. The conditioned medium (100μl) of each different treatment was collected and transferred to microplate wells where the human VEGF level was determined with a VEGF ELISA kit (RayBio) following the manufacturer's instruction. VEGF protein concentration in the medium was determined colormetrically using a micro-plate reader (BioTek) at 450 nm.

### Angiogenesis assay *in vitro*

Human umbilical vein endothelial cells (HUVEC) were used to evaluate the tube forming ability of endothelial cells induced by secreted VEGF in conditioned medium. Fifty μl ECMatrixTM Gel (*In Vitro* Angiogenesis Assay Kit, Merck Millipore) were loaded in each well of a 96-well plate and the HUVECs (10^4^cells/100μl) were seeded on the top of ECMatrix layer in each well. Then 100μl of conditioned medium from each different treatment was loaded and the plates were incubated at 37^°^C, 5% (v/v) CO_2_ for 6 hours. Recombinant human VEGF (10ng/ml) was used as positive control and each sample was loaded in triplicate. The cell-tubes were visualized by adding 50μl of 2% MTT for 10 minutes at room temperature and quantified under an inverted light microscope at 40 × magnification.

### Dual-luciferase^®^reporter (DLR™) assay

Three DNA segments based on human VEGF promoter sequence [49] (gi|224589818:43727945-43737944 Homo sapiens chromosome 6, GRCh37.p10 Primary Assembly) were designed and synthesised. Main truncated promoter-region (WT) contained 5′flanking sequences extending -805 nucleotides from the transcriptional start site and including two PPREs (−796 to −790bp and −443 to −437bp) [29]. *PPRE* sequence (AGGCCA) [50] in both locations was mutated (ATGCAT) in the second DNA fragment. In the third DNA fragment, the sequence was shortened to −393bp and both PPREs were deleted. These DNA fragments were ligated into pGL3 Luciferase Reporter Vectors (Promega, WI, USA) to form 3 reporter constructs named WT, M1 and M2, respectively. Prostate cancer cell lines were cultured and then seeded into 24-well plates (5×10^4^cells/well in 1ml medium). Cancer cells were transiently co-transfected with *Renilla* luciferase plasmid plus different luciferase constructs. The luciferase activities were measured using DLR™ Assay (Promega, WI, USA).

### Statistical analysis

Student's t-test was used to evaluate the difference in means between two groups for proliferation, invasion, in soft agar, *in vitro* angiogenesis assay, as well as average tumour volumes in *in vivo* assay and difference in luciferase levels in DLR assay. A value of *p* < 0.05 was defined as statistically significant. Correlation between the PPARγ expression and the sizes of primary tumors produced in nude mice was assessed by χ^2^ analysis.

## References

[1] Kurahashi N, Inoue M, Iwasaki M, Sasazuki S, Tsugane AS (2008). Japan Public Health Center-Based Prospective Study G. Dairy product, saturated fatty acid, and calcium intake and prostate cancer in a prospective cohort of Japanese men. Cancer Epidemiol Biomarkers Prev.

[2] Chavarro JE, Stampfer MJ, Campos H, Kurth T, Willett WC, Ma J (2008). A prospective study of trans-fatty acid levels in blood and risk of prostate cancer. Cancer Epidemiol Biomarkers Prev.

[3] Hotamisligil GS (2006). Inflammation and metabolic disorders. Nature.

[4] Zimmerman AW, Veerkamp JH (2002). New insights into the structure and function of fatty acid-binding proteins. Cell Mol Life Sci.

[5] Madsen P, Rasmussen HH, Leffers H, Honore B, Celis JE (1992). Molecular cloning and expression of a novel keratinocyte protein (psoriasis-associated fatty acid-binding protein [PA-FABP]) that is highly up-regulated in psoriatic skin and that shares similarity to fatty acid-binding proteins. J Invest Dermatol.

[6] Masouye I, Saurat JH, Siegenthaler G (1996). Epidermal fatty-acid-binding protein in psoriasis, basal and squamous cell carcinomas: an immunohistological study. Dermatology.

[7] Celis A, Rasmussen HH, Celis P, Basse B, Lauridsen JB, Ratz G, Hein B, Ostergaard M, Wolf H, Orntoft T, Celis JE (1999). Short-term culturing of low-grade superficial bladder transitional cell carcinomas leads to changes in the expression levels of several proteins involved in key cellular activities. Electrophoresis.

[8] Sinha P, Hutter G, Kottgen E, Dietel M, Schadendorf D, Lage H (1999). Increased expression of epidermal fatty acid binding protein, cofilin, and 14-3-3-sigma (stratifin) detected by two-dimensional gel electrophoresis, mass spectrometry and microsequencing of drug-resistant human adenocarcinoma of the pancreas. Electrophoresis.

[9] Liu RZ, Graham K, Glubrecht DD, Germain DR, Mackey JR, Godbout R (2011). Association of FABP5 expression with poor survival in triple-negative breast cancer: implication for retinoic acid therapy. Am J Pathol.

[10] Barbus S, Tews B, Karra D, Hahn M, Radlwimmer B, Delhomme N, Hartmann C, Felsberg J, Krex D, Schackert G, Martinez R, Reifenberger G, Lichter P (2011). Differential retinoic acid signaling in tumors of long- and short-term glioblastoma survivors. J Natl Cancer Inst.

[11] Jing C, Beesley C, Foster CS, Rudland PS, Fujii H, Ono T, Chen H, Smith PH, Ke Y (2000). Identification of the messenger RNA for human cutaneous fatty acid-binding protein as a metastasis inducer. Cancer Res.

[12] Jing C, Beesley C, Foster CS, Chen H, Rudland PS, West DC, Fujii H, Smith PH, Ke Y (2001). Human cutaneous fatty acid-binding protein induces metastasis by up-regulating the expression of vascular endothelial growth factor gene in rat Rama 37 model cells. Cancer Res.

[13] Adamson J, Morgan EA, Beesley C, Mei Y, Foster CS, Fujii H, Rudland PS, Smith PH, Ke Y (2003). High-level expression of cutaneous fatty acid-binding protein in prostate carcinomas and its effect on tumorigenicity. Oncogene.

[14] Bao Z, Malki MI, Forootan SS, Adamson J, Forootan FS, Chen D, Foster CS, Rudland PS, Ke Y (2013). A novel cutaneous Fatty Acid-binding protein-related signaling pathway leading to malignant progression in prostate cancer cells. Genes Cancer.

[15] Morgan EA, Forootan SS, Adamson J, Foster CS, Fujii H, Igarashi M, Beesley C, Smith PH, Ke Y (2008). Expression of cutaneous fatty acid-binding protein (C-FABP) in prostate cancer: potential prognostic marker and target for tumourigenicity-suppression. Int J Oncol.

[16] Mangelsdorf DJ, Thummel C, Beato M, Herrlich P, Schutz G, Umesono K, Blumberg B, Kastner P, Mark M, Chambon P, Evans RM (1995). The nuclear receptor superfamily: the second decade. Cell.

[17] Lemberger T, Desvergne B, Wahli W (1996). Peroxisome proliferator-activated receptors: a nuclear receptor signaling pathway in lipid physiology. Annu Rev Cell Dev Biol.

[18] Mansure JJ, Nassim R, Chevalier S, Szymanski K, Rocha J, Aldousari S, Kassouf W (2013). A novel mechanism of PPAR gamma induction via EGFR signalling constitutes rational for combination therapy in bladder cancer. Plos One.

[19] Sikka S, Chen L, Sethi G, Kumar AP (2012). Targeting PPARgamma signaling cascade for the prevention and treatment of prostate cancer. PPAR Res.

[20] Forootan FS, Forootan SS, Malki MI, Chen D, Li G, Lin K, Rudland PS, Foster CS, Ke Y (2014). The expression of C-FABP and PPARgamma and their prognostic significance in prostate cancer. Int J Oncol.

[21] Wang M, Wise SC, Leff T, Su TZ (1999). Troglitazone, an antidiabetic agent, inhibits cholesterol biosynthesis through a mechanism independent of peroxisome proliferator-activated receptor-gamma. Diabetes.

[22] Hanahan D, Weinberg RA (2000). The hallmarks of cancer. Cell.

[23] Forootan SS, Bao ZZ, Forootan FS, Kamalian L, Zhang Y, Bee A, Foster CS, Ke Y (2010). Atelocollagen-delivered siRNA targeting the FABP5 gene as an experimental therapy for prostate cancer in mouse xenografts. Int J Oncol.

[24] Rohrl C, Kaindl U, Koneczny I, Hudec X, Baron DM, Konig JS, Marian B (2011). Peroxisome-proliferator-activated receptors gamma and beta/delta mediate vascular endothelial growth factor production in colorectal tumor cells. J Cancer Res Clin Oncol.

[25] Bishop-Bailey D (2011). PPARs and angiogenesis. Biochem Soc Trans.

[26] Kristiansen G, Jacob J, Buckendahl AC, Grutzmann R, Alldinger I, Sipos B, Kloppel G, Bahra M, Langrehr JM, Neuhaus P, Dietel M, Pilarsky C (2006). Peroxisome proliferator-activated receptor gamma is highly expressed in pancreatic cancer and is associated with shorter overall survival times. Clin Cancer Res.

[27] Biscetti F, Gaetani E, Flex A, Aprahamian T, Hopkins T, Straface G, Pecorini G, Stigliano E, Smith RC, Angelini F, Castellot JJ, Pola R (2008). Selective activation of peroxisome proliferator-activated receptor (PPAR)alpha and PPAR gamma induces neoangiogenesis through a vascular endothelial growth factor-dependent mechanism. Diabetes.

[28] Eisermann K, Broderick CJ, Bazarov A, Moazam MM, Fraizer GC (2013). Androgen up-regulates vascular endothelial growth factor expression in prostate cancer cells via an Sp1 binding site. Mol Cancer.

[29] Peeters LL, Vigne JL, Tee MK, Zhao D, Waite LL, Taylor RN (2005). PPAR gamma represses VEGF expression in human endometrial cells: implications for uterine angiogenesis. Angiogenesis.

[30] Gonzalez-Moreno O, Lecanda J, Green JE, Segura V, Catena R, Serrano D, Calvo A (2010). VEGF elicits epithelial-mesenchymal transition (EMT) in prostate intraepithelial neoplasia (PIN)-like cells via an autocrine loop. Exp Cell Res.

[31] Stoner M, Wormke M, Saville B, Samudio I, Qin C, Abdelrahim M, Safe S (2004). Estrogen regulation of vascular endothelial growth factor gene expression in ZR-75 breast cancer cells through interaction of estrogen receptor alpha and SP proteins. *Oncogene*.

[32] Chung LW, Chang SM, Bell C, Zhau H, Ro JY, von Eschenbach AC (1988). Prostate carcinogenesis evoked by cellular interaction. Environ Health Perspect.

[33] Condon MS, Kaplan LA, Crivello JF, Horton L, Bosland MC (1999). Multiple pathways of prostate carcinogenesis analyzed by using cultured cells isolated from rats treated with N-methyl-N-nitrosourea and testosterone. Mol Carcinog.

[34] Ling MT, Chan KW, Choo CK (2001). Androgen induces differentiation of a human papillomavirus 16 E6/E7 immortalized prostate epithelial cell line. J Endocrinol.

[35] Reid AH, Attard G, Barrie E, de Bono JS (2008). CYP17 inhibition as a hormonal strategy for prostate cancer. Nat Clin Pract Urol.

[36] Asangani IA, Dommeti VL, Wang X, Malik R, Cieslik M, Yang R, Escara-Wilke J, Wilder-Romans K, Dhanireddy S, Engelke C, Iyer MK, Jing X, Wu YM, Cao X, Qin ZS, Wang S (2014). Therapeutic targeting of BET bromodomain proteins in castration-resistant prostate cancer. Nature.

[37] Bubendorf L, Kononen J, Koivisto P, Schraml P, Moch H, Gasser TC, Willi N, Mihatsch MJ, Sauter G, Kallioniemi OP (1999). Survey of gene amplifications during prostate cancer progression by high-throughout fluorescence in situ hybridization on tissue microarrays. Cancer Res.

[38] Chen CD, Welsbie DS, Tran C, Baek SH, Chen R, Vessella R, Rosenfeld MG, Sawyers CL (2004). Molecular determinants of resistance to antiandrogen therapy. Nat Med.

[39] Visakorpi T, Hyytinen E, Koivisto P, Tanner M, Keinanen R, Palmberg C, Palotie A, Tammela T, Isola J, Kallioniemi OP (1995). In vivo amplification of the androgen receptor gene and progression of human prostate cancer. Nat Genet.

[40] Baylin SB, Jones PA (2011). A decade of exploring the cancer epigenome - biological and translational implications. Nat Rev Cancer.

[41] Jiang Q, Yeh S, Wang X, Xu D, Zhang Q, Wen X, Xia S, Chang C (2012). Targeting androgen receptor leads to suppression of prostate cancer via induction of autophagy. J Urol.

[42] Nualart F, Garcia MDLA, Medina RA, Owen GI (2009). Glucose Transporters in Sex Steroid Hormone Related Cancer. Curr Vasc Pharmacol.

[43] Katzenwadel A, Wolf P (2015). Androgen deprivation of prostate cancer: Leading to a therapeutic dead end. Cancer Lett.

[44] Sramkoski RM, Pretlow TG, Giaconia JM, Pretlow TP, Schwartz S, Sy MS, Marengo SR, Rhim JS, Zhang D, Jacobberger JW (1999). A new human prostate carcinoma cell line, 22Rv1. In Vitro Cell Dev Biol Anim.

[45] Kaighn ME, Narayan KS, Ohnuki Y, Lechner JF, Jones LW (1979). Establishment and characterization of a human prostate carcinoma cell line (PC-3). Invest Urol.

[46] Janik P, Briand P, Hartmann NR (1975). The effect of estrone-progesterone treatment on cell proliferation kinetics of hormone-dependent GR mouse mammary tumors. Cancer Res.

[47] Foster CS, Gosden CM, Ke YQ (2006). Primer: tissue fixation and preservation for optimal molecular analysis of urologic tissues. Nature clinical practice Urology.

[48] Remmele W, Stegner HE (1987). Recommendation for uniform definition of an immunoreactive score (IRS) for immunohistochemical estrogen receptor detection (ER-ICA) in breast cancer tissue [Article in German]. Pathologe.

[49] Mueller MD, Vigne JL, Minchenko A, Lebovic DI, Leitman DC, Taylor RN (2000). Regulation of vascular endothelial growth factor (VEGF) gene transcription by estrogen receptors alpha and beta. Proc Natl Acad Sci U S A.

[50] Venkatachalam G, Kumar AP, Sakharkar KR, Thangavel S, Clement MV, Sakharkar MK (2011). PPARgamma disease gene network and identification of therapeutic targets for prostate cancer. J Drug Target.

